# Inhibition of nuclear factor-kappa B enhances the tumor growth of ovarian cancer cell line derived from a low-grade papillary serous carcinoma in p53-independent pathway

**DOI:** 10.1186/s12885-016-2617-2

**Published:** 2016-08-02

**Authors:** Xue Xiao, Gong Yang, Peng Bai, Shunping Gui, Tri M. Bui Nyuyen, Imelda Mercado-Uribe, Mei Yang, Juan Zou, Qintong Li, Jianguo Xiao, Bin Chang, Guangzhi Liu, He Wang, Jinsong Liu

**Affiliations:** 1 Department of Gynecology and Obstetrics, West China Second University Hospital, Sichuan University, Chengdu, 610041 People’s Republic of China; 2 West China School of Preclinical and Forensic Medicine, Sichuan University, Chengdu, 610041 People’s Republic of China; 3 Cancer Research Laboratory, Fudan University Shanghai Cancer Center, Shanghai, 200032 China; 4 Department of Oncology, Shanghai Medical College, Fudan University, Shanghai, 200032 China; 5 Department of Pathology, University of Texas M. D. Anderson Cancer Center, Houston, TX USA; 6 Department of Pathology, West China Second University Hospital, Sichuan University, Chengdu, 610041 People’s Republic of China; 7 Department of Biochemistry and Molecular Biology, George Washington University, Washington, D.C., USA; 8 Department of Pathology, Shihezi University School of Medicine, Shihezi, Xinjiang 82002 China

**Keywords:** Ovarian cancer, NF-kB, Pro-apoptosis, Tumor suppressor, IkBαM

## Abstract

**Background:**

NF-kB can function as an oncogene or tumor suppressor depending on cancer types. The role of NF-kB in low-grade serous ovarian cancer, however, has never been tested. We sought to elucidate the function of NF-kB in the low-grade serous ovarian cancer.

**Methods:**

The ovarian cancer cell line, HOC-7, derived from a low-grade papillary serous carcinoma. Introduction of a dominant negative mutant, IkBαM, which resulted in decrease of NF-kB function in ovarian cancer cell lines. The transcription ability, tumorigenesis, cell proliferation and apoptosis were observed in derivative cell lines in comparison with parental cells.

**Results:**

Western blot analysis indicated increased expression of the anti-apoptotic proteins Bcl-xL and reduced expression of the pro-apoptotic proteins Bax, Bad, and Bid in HOC-7/IĸBαM cell. Further investigations validate this conclusion in KRAS wildtype cell line SKOV3. Interesting, NF-kB can exert its pro-apoptotic effect by activating mitogen-activated protein kinase (MAPK) phosphorylation in SKOV3 ovarian cancer cell, whereas opposite changes detected in p-MEK in HOC-7 ovarian cancer cell, the same as some chemoresistant ovarian cancer cell lines. In vivo animal assay performed on BALB/athymic mice showed that injection of HOC-7 induced subcutaneous tumor growth, which was completely regressed within 7 weeks. In comparison, HOC-7/IĸBαM cells caused sustained tumor growth and abrogated tumor regression, suggesting that knock-down of NF-kB by IĸBαM promoted sustained tumor growth and delayed tumor regression in HOC-7 cells.

**Conclusion:**

Our results demonstrated that NF-kB may function as a tumor suppressor by facilitating regression of low grade ovarian serous carcinoma through activating pro-apoptotic pathways.

## Background

NF-kB has long been recognized as a potent tumor promoter and a lot drugs are targeting NF-kB in cancer treatment. However, more recent evidence suggests that NF-kB can also inhibit the tumor growth. The first pieces of evidence that NF-kB can function as a tumor suppressor came from studies of chemically-induced skin cancer, in which inhibition of NF-kB via IĸB-SR expression in keratinocytes enhanced the multiplicity of squamous cell carcinomas in response to 7,12-Dimethylbenz(a)anthracene (DMBA). Furthermore, inhibition of NF-kB in primary human keratinocytes promoted Ras-mediated transformation. The tumor suppressing activity of NF-kB was explained either by inhibition of cell cycle or by downregulating of MAPKs pathway [1–3]. Members from distinct signaling cascades of the MAPK family such as SAPK/JNK, p38 MAPK, and ERK1/2 are known to determine cell fate during DNA damage, mitogenic stimuli, and survival. Nevertheless, in different cell line and under different irritation, the involved signaling cascades of MAPKs are different [1, 4–7]. In particular, the interplay between NF-kB and JNK may play a critical role in the development of Den- induced hepatocellular carcinoma (HCC), while ERK activation was seen in Ras-IĸBα tumors and patient’s squamous cell carcinoma (SCCs).

Our previous study showed that in ovarian carcinoma cell lines OVCA433 and OVCR3, introduction of a dominant negative mutant IĸBα (IĸBαM), which constitutively suppresses NF-kB function, resulted in cell proliferation and inhibition of apoptosis through ERK/MAPKs pathway. Despite the solid evidence supporting the potential relevance of both activation of Ras/MAPK pathway and blockade of NF-kB in at least a subset of spontaneous human epidermal SCCs, the exact functions latent mechanism of NF-kB and MAPK pathways in low grade ovarian cancer are still unknown. The KRAS and BRAF are the upstream of MAPKs pathway and genetic alterations in these genes are associated with carcinogenesis [8–11]. Because therapeutics interventions inhibiting Ras and NF-kB pathways are being developed to treat human ovarian cancer, it is crucial to assess the effects of altering these regulators. Since the quantities of active Ras-GTP were comparable in Ras-IĸBα tumors and in human epithelial cancer cells with defined Ras mutations [12, 13], it is not hard to propose that the tumorigenesis was not due to expression of more active Ras than is expressed by endogenous mutant alleles. The mechanism about Ras and its phophorylated MAPK targets and the activity of NF-kB were uncertain, however, by critical shortage of ras mutation cell line and mouse model. Excitedly, HOC-7 provides us a kras mutation model to learn about the potential mechanism of these factors.

The origin, development and outcome of low-grade ovarian cancer are different from high-grade ovarian cancer. P53 overexpression and mutations are infrequent in low-grade ovarian cancer but occur in as many as 50 to 80 % of high-grade ovarian cancer. Depending on the morphologic, immunohistochemical and molecular genetic analyses of p53 comparing with low- and high-grade ovarian cancer, functional mutations, defined as mutations leading to the alteration of the structure of encoded protein, were detected in 50.8 % of high-grade cancer and 8.3 % of low-grade cancer. In our study, the role of p53 in NF-kB-related tumor suppression was also mentioned.

In this study, we investigated the functional role of NF-kB in cancer development using ovarian cancer as a model system. Our study provides first experimental evidence that NF-kB may inhibit the HOC-7 tumorigenesis in a p53 independent signaling manner.

## Methods

### Cell lines, media and plasmids

HOC-7 with an average doubling time of 16.4 days is a kind gift from Dr R. N. Buick (University of Arizona, Tucson), which was derived from ascites of a patient with a well-differentiated Stage III serous adenocarcinoma of the ovary [9, 14]. SKOV3 human ovarian cancer cell lines were bought from the American Type Culture Collection (Rockville, MD). HOC-7 and SKOV3 cell lines were cultured in Minimum Essential Alpha 1×/ RPMI-1640 medium (GIBCO BRL, Life Technologies, Gaithersburg, MD) supplemented with 10 % fetal bovine serum (HyClone, Logan, UT) at 37 °C with 5 % CO_2_. Both HOC-7 and SKOV3 were transfected with dominant negative mutant IĸB, henceforth IĸBαM, using N-[1-(2,3-dioleoyloxyl)propyl]-N,N,N-trimethylammoniummethyl sulfate liposomal transfection reagent (Boehringer Mannheim, Indianapolis, IN) and selected by neomycin antibiotics (2.5ug/ml) for 14 days. The plasmid used for stable transfection was a pBabe-U6/puromycin vector (5.4 kb) containing IkBαM under cytomegalovirus (CMV) promoter expression control. The primers used to sequence IkBαM were p1: catatggatccatgtttcagccagctgggcacg p2: cctatctcgagttataatgtcagacgctggcctc. The mutation place in the serines 32 and 36 of IkBα were replaced by glycine and alanine, which damaged the phosphorylation and followed proteolysis, eventually led to the irreversible binding to Rel/NF-kB and inhibited its translocation in nuclei [9, 15–18]. In addition, we transfected HOC-7 and IkBαM expressing HOC-7 cell line with p53 siRNA, the positive colony were selected by puromycin (300ug/ml). SiRNAs against p65 and p53 and control siRNA were purchased from Santa Cruz Biotechonology.

### Mutantional analysis of KRAS in HOC-7 and SKOV3 cell lines

Genomic DNA was purified from all of the HOC-7 and SKOV3 cell lines using the QIAGEN’s DNeasy Tissue Kit (QIAGEN Inc, Chatsworth, CA, USA). PCR was performed ensued by nucleotide sequencing using the iCycler (Bio-Rad, Hercules, CA, USA), which is including the exon 1 of KRAS. The primers used to sequence were: 5-taaggcctgctgaaaatgactg-3 (forward); 5-tggtcctgcaccagtaatatgc-3 (reverse); and 5-ctgcaccagtaatatgcatattaaaac-3 (sequencing); The thermal profile was: first denaturation step 95 °C 2 min, followed by 95 °C 30 s, 58 °C for 30s, 72 °C for 35 s, total 35 cycles; 72 °C for 10 min [19, 20]. The Lasergene program (DNASTAR, Madison, WI) was used to analyze these sequences.

### Electrophoretic mobility shift assay (EMSA) and luciferase reporter assay

The electrophoretic mobility shift assay was performed as described before. Briefly, the nuclear extracts of different cell lines were prepared by lysed in nuclear extract buffer (20 mM HEPES, 400 mM NaCl, 1 Mm EDTA, 1 Mm EGTA, 10 μM dithiothreitol, 20 μg/ml leupeptin, 20 μg/ml aprotinin, 500 μg/ml benzamidine) and the concentrations were determined using the Coomassie brilliant blue G250 assay kit. Double-stranded deoxyoligonucleotides containing the NF-kB consensus recognition site (5′-AGT TGA GGG GAC TTT CCC AGG C-3′ and 5′-GCC TGG GAA AGT CCC CTC AAC T-3′, Santa Cruz Biotechnology Inc, Santa Cruz, CA) were labeled with (r-^32^p)ATP using T4 polynucleotide kinase according to the protocol. The nuclear probe was incubated with the radiolabeled probe DNA (3.5pmol, 10 μCi) and 4 μl 5 × binding buffer. For the supershift, the proteins were incubated with p65 or p50 monoclonal antibody before ^32^p labeled. All of the DNA-protein complexes were resolved on 5 % PAGE in Tris/glycine buffer after incubated at room temperature for 30 min. For the NF-kB transcription assay, seeded cells were precultured to 75 % confluence and transfected with and HIV promoter-driven luciferase cDNA plasmid, and then tested according to promega kit.

### Cell proliferation assay

Cell proliferation was determined by examined by 3-[4, 5-dimethylthiazol-2-yl]-2, 5-diphenyltetrazolium bromide assay (MTTassay; Sigma) with the CellTiler 96 Aqucous One Solution Cell Proliferation Assay Kit (Promega) according to the manufacturer’s protocol. Four different cell lines HOC-7, HOC-7 IĸBαM, HOC-7 P53i and HOC-7 IĸBαM P53i were seeded at 5 × 10^3^ cells/well in 96-well tissue culture plates. Cell growth curve was assessed every 24 h and totally 3 days.

### Soft agar colony formation assay

Soft agar growth assay was performed as previously described (Difco, Detroit, MI). The four cell lines were mixed with 0.4 % agar were overlaid above the supporting 0.6 % agar and cultured total 25 days with replenished medium. Random fields were recorded using a Leica TCS4D confocal scanning laser microscope (Leica, Solms, Germany) and the total number and the size of the colonies were counted.

### Western blot analysis

Western blot analysis was performed as preciously described. Lysates of treated cells or tumor samples were collected and lysed in radioimmunoprecipitation assay buffer. Equal amounts of proteins were resolved on SDS-PAGE and then transferred onto a polyvinylidene difluoride membrane. Specific primary antibodies were used for the following molecules : IĸBα(Cabiochem, San Diego, CA, USA), p65, Iĸĸα, Iĸĸβ, AKT, p-AKT, MEK, p-MEK, ERK, p-ERK, p38, caspase-10, caspase-1, cyclinA, cyclinB, PUMA, BID, CIAP, Bad, p-Bad, PKC (Cell signaling Technology), JNK2/1, p-JNK, TSP1, Bcl-2, Bcl-xL, Bax, APAF1, Fas, caspase-8, Mrg1, cyclinD, p53, MMP1 (Santa Cruze Biotechnology, CA), caspase-9, caspase-3 (BioLegend). β-Actin (Santa Cruze Biotechnology, CA) was used to assess the equal loading. The phosphorylated protein controls obtained from blots that had been stripped and re-probed for the same sample lane. Sixty microgram of cell protein extract was loaded per lane. CS-710 Calibrated Imaging Densitometer (Bio-Rad) was utilized for densitometric quantification.

### Apoptosis detection

Derivative cells and control cells were cultured to 80 % confluence. Cells were harvested after being washed three times with ice-cold phosphate-buffered saline (PBS). Subsequently, cells were labeled with Annexin V and propidium iodide according to the manufacturer’s protocol (BD ApoAlert Annexin V-FITC Apoptosis kit; BD Biosciences, PaloAlto, CA) [21]. The percentage of apoptotic cells was determined by (M2) peak in the histogram generated by FACSCalibur system and Cell Quest software (BD Biosciences).

### Tumor xenograft in nude mice

The xenograft tumor model has been described earlier [22]. Briefly, 5 × 106 cells of either wild type HOC-7, SKOV3, IĸBαM expressing HOC-7 and SKOV3 or P53-inactivating cells were subcutaneously injected into 4- to 6-week-old female BALB/athymic nude mice (NCI-Frederick, NIH). Each group includes 8 mice. One week after implantation, the mice were checked every 2 days per week. The control group received saline (PBS) only. The same quantity of saline was used in these groups to resuspend different cell lines. General clinical observation of the mice included determination of syndrome, side effect and body weight. To determine tumor size, we measured two perpendicular diameters of the xenograft in centimeters by calibers. Tumor weight was then estimated using formula 1/2a × b2, where a is the long diameter and b is the short diameter [23, 24]. 66 days after the first injection, all mice were euthanized by carbon dioxide asphyxiation and the tumor tissues were processed for immunohistochemistry staining followed by 10 % formalin fixation overnight. All protocols for animal use were reviewed and approved by the Animal Care Committee of West China Second University hosipital in accordance with Institutional Animal Care and Use Committee guidelines.

### Patient tissue specimens

The use of tissue blocks and chart reviews were approved by the Institutional Review Board of Sichuan University. The specimens, including controls, used in our study were preserved and stored by the Tissue Bank Core Facility at Sichuan University. Detailed quality control procedures for tissue sampling were chosen and implemented by the Tissue Bank Core Facility at Sichuan University. Patients had been treated with either chemotherapy or irradiation by the treating physicians, and the selection of patient tissues was not based on the treatments. Follow up information was updated through March 2011 by reviewing medical records. The randomly selected formalin-fixed paraffin-embedded tissues included normal ovarian tissues (*n* = 20) and low-grade serous ovarian carcinomas (*n* = 416) were not matched. Tumor sample collection and tissue microarray construction have been described previously [25, 26]. Briefly, ovarian tissue microarray blocks were diagnosed in duplicate by two gynecologic pathologists who reviewed hematoxylin and eosin–stained sections and constructed by taking core samples from morphologically representative areas of paraffin-embedded tumor tissues and assembling them on a recipient paraffin block. The follow-up protocol have been mentioned previously, the same as the characteristics of exclusion criteria and eligible participants. All of the participants provided written informed consent and the study protocol was agreed by the institutional review boards of Sichuan University (no.11789).

### Immunohistochemical staining and analysis

The streptavidin-peroxidase immunohistostaining method for NF-kB p65 was performed as described elsewhere [27, 28]. Briefly, samples were fixed in 10 % formalin buffer and embedded in paraffin. Tissue sections (4 μm thick) were steamed in universal decloaker (Biocare Medical, Walnut Creek, CA, USA) for antigen retrieval, followed by 19 min protein-blocking (Biocare Medical). All slides were first incubated against NF-kB p65 (1:500, for 1 h at room temperature; Santa Cruz Biotechnology, Inc., Santa Cruz, CA, USA) and then treated with secondary antibody (Biocare Medical) and horseradish peroxidase for 15 min each. The tissues were stained for 3 min with high sensitivity 3,3′-diaminobenzidine tetrahydrochloride, counterstained with hematoxylin, dehydrated and then mounted [29, 30].

### Statistical analysis

Statistical analysis was performed by using Fisher’s exact test at different time points for the mean tumor sizes of each group. X-tile software was used to find the optimal cut-off point. Monte Carlo simulations was used to determine the prognostic significance. Disease-specific survival rates were calculated by the Kaplan-Meier method and compared by the log-rank test. Cox proportional hazards regression models in Statistica software (SAS Institute, SAS Language Reference, version 8, SAS Institute, Inc) were used for multivariate analyses of survival. The rest of statistical analysis was performed by using Student’s t-test (STATISTICA6 software, StatSoft, Tulsa, OK). Differences with *P-*value was less than 0.05 were considered significant. All statistical tests were two-sided.

## Results

### KRAS mutation exists in HOC-7 ovarian cancer cell lines and human low grade serous ovarian cancers

**Fig. 1:**
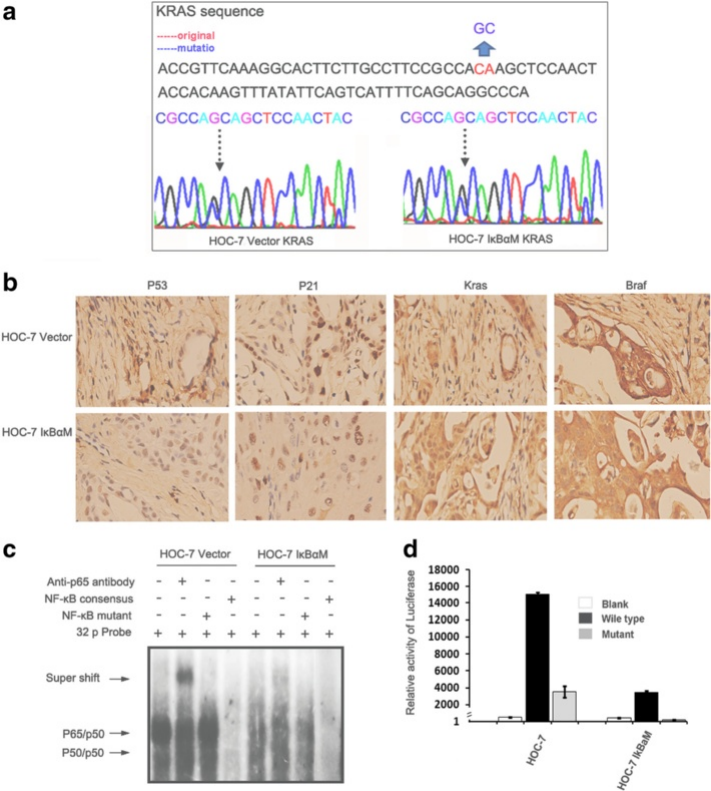
Genetic specific of ovarian cancer cells. **a** Sequence of KRAS gene in HOC-7 and relative transgenic cells. **b** The expression of kras, braf, p21 and p53 in HOC-7 and relative transgenic tumor tissues by immunohistochemical staining (400×). **c** Electrophoretic mobility shift assays showing that the binding of NF-kB to its promoter DNA consensus sequence was reduced in IkBαM-transfected cells. **d** Luciferase-reporter assays showing the reduced transcriptional activity of NF-kB. Error bars =95 % confidence intervals. Blank represents the empty control vector, wild type represents the luciferase reporter vector, which has an HIV promoter containing binding sites to NF-kB protein. The intensity of luciferase activity thus represents the cellular NF-kB activity. Mutant represents the luciferase reporter vector with NF-kB binding sites eliminated. Ectopic expression of IkBαM markedly reduced the reporter activity, indicative of dampened cellular NF-kB activity

Ovarian cancer cell lines HOC-7 and transformed cells were first analysed for mutations in the KRAS gene. As shown in Fig. 1a, KRAS exon 1 mutation (from ACA to AGC) were initially screened by PCR-SSCP and then confirmed by direct sequencing. Xenograft tumors produced by subcutaneous injection of HOC-7, HOC-7 IĸBαM cell lines were evaluated by histopathology. Slides showed similar morphology as low-grade human serous ovarian carcinoma. Following immunohistochemical staining showed that HOC-7 and HOC-7 IĸBαM tumor tissues expressing KRAS strongly due to persistent activity of KRAS/MAPKs pathway, which confirm the KRAS mutation (Fig. 1b).

### Inhibition of constitutive activation of NF-kB in HOC-7 carrying IĸBα

To study the importance of NF-kB pathway in tumorigenesis, we selectively blocked NF-kB activation by targeted expression of a super-repressor form of IĸBα. In EMSA assessment of nuclear extracts from both transfected and parental HOC-7 cells, IĸBα-transfected cells showed reduced DNA binding and gene transcription activity of NF-kB compared with parental cells (Fig. 1c-d). Western blot showed overexpression of IkBα and decreased expression of Ikkα and p65 in nuclei of HOC-7 IĸBαM cells. As shown in Fig. 4a, clear bands were shown in expected 37 kDa size, which represent endogenous IĸBα. Interesting, an extra band was appeared at a little bit higher size (40 kDa) in IĸBαM transfected HOC-7 cells, but not in parental HOC-7 cells. To further test the inhibiting function of IkBαM in NF-kB mediated gene expression, downstream markers of NF-kB were measured using western blotting. As expected, IkBαM repressed expression of PUMA logged in NF-kB downstream pathway, indicating that IkBαM was stably expressed in HOC-7 cells and inhibited the constitutive function of NF-kB, as shown in Fig. 4b.

### Consitutive activation of NF-kB inhibits tumor growth in ovarian cancer cell lines

**Fig. 2:**
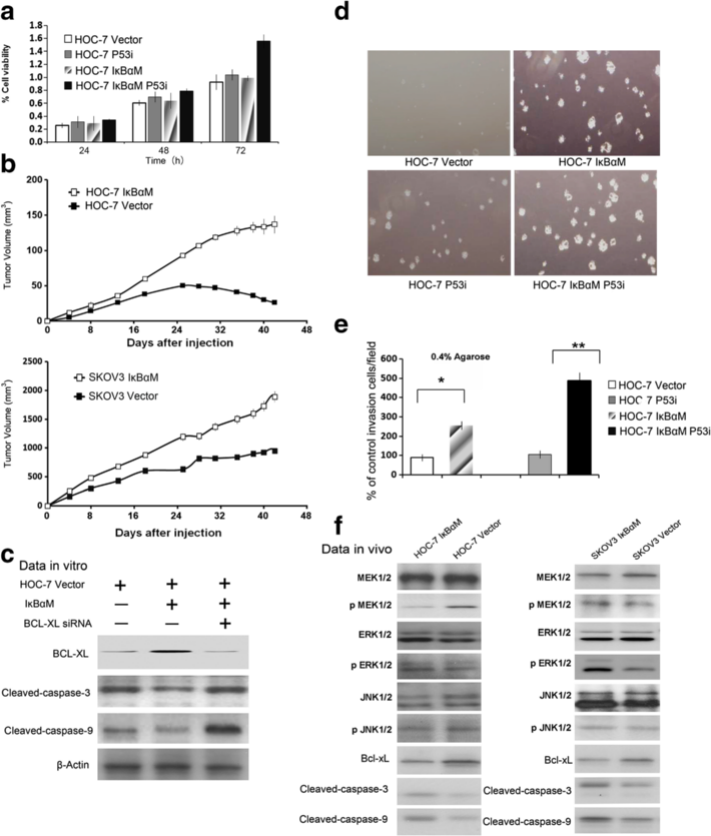
NF-kB induced tumor growth in HOC-7 cells. **a** HOC-7-IkBαM cells displayed dramatically increase of cell proliferation compare with parental cells. **b** colony formation of HOC-7 transgenic cells and parental cells. **c** In vivo, NF-kB induced tumor growth both in SKOV3 and HOC-7 xenograft nude mice“ to ”Fig. 2. NF-kB induced tumor growth in HOC-7 cells. **a** HOC-7-IkBαM cells displayed dramatically increase of cell proliferation compare with parental cells. **b** In vivo, NF-kB induced tumor growth both in SKOV3 and HOC-7 xenograft nude mice (**c**) BCL-xl is the key protein involved in the phenotypic shift. **d** colony formation of HOC-7 transgenic cells and parental cells. **e** Data represent the mean ± standard error of the mean (SEM) percentage of the number of invasion cells compared to the control; *p < 0.05, *n* = 3. Error bars = 95 % confidence intervals. **f** NF-kB - induced apoptosis correlated with MAPKs pathway and apoptosis pathway in vivo. The western blot data from the mice tumors showed that expression of p-MEK increased in mice tissue carrying HOC-7 IkBαM compare with those carrying HOC-7 vector, while the expression of p-ERK instead of p-MEK decreased in mice tissue carrying SKOV3 IkBαM compare with those carrying SKOV3 vector. Generally, NF-kB induced apoptosis in a MAPKs dependent pathway in vivo

To compare the cell growth rate between parental cells with constitutive NF-kB activation and transgenic cells with stably expression of IĸBαM, we tested its effect on tumor growth in two ovarian cancer cell lines, SKOV3 and HOC-7. As shown in Fig. 2a, HOC-7 carrying IkBαM resulted in 48 % cell growth increase compared with parental cells. Additionally, prolonged culture of cells vastly increased this growth differences. Furthermore, HOC-7 cells carrying IkBαM showed 2.4 times increase anchorage independent growth on soft agar compared with parental cells, which exhibit dramatic colony-forming capacity (Fig. 2d-e). These data indicated that block of NF-kB activity by IkBαM could effectively induce growth and migration ability in ovarian epithelial cells.

### NF-kB inhibits the tumor growth in BALB/athymic nude mice

To investigate if inhibition NF-kB activity can induce tumor growth in vivo, we first established a xenograft model of HOC-7 IkBαM and SKOV3 IkBαM cells in BALB/athymic nude mice using parental cells as control. All of the mice given i.p. injection developed tumor showed for lethargy, poor appetite and abdominal enlargement. Injection of HOC-7 into subcutis of nude mice led to tumor growth up to one week followed by complete regression at the end of 66 days. In contrast, subcutaneous injection of HOC-7 expressing IĸBαM resulted in tumor growth for up to five weeks followed by a steeply increase for 4 weeks (from 38 days to 66 days). At the 38 days of experiments, the subcutaneous tumor growth in 8 mice inoculated with HOC-7-IkBαM increased 82 % tumor weight as compared with that in HOC-7 control mice. 87 % increase of s.c tumor volume in HOC-7-IkBαM was observed (Fig. 2b). Similarly, nude mice injection with IĸBαM-transfected SKOV3 had more subcutaneous tumor growth than those injected with parental cells. The expression of IĸBαM promotes tumor growth and abrogates tumor regression. Interesting, the western blot data of IkBαM expression of mice tumors in Fig. 2f indicated that IkBαM expression correlate with MAPKs pathway and apoptosis pathway, which coincided with the data in vitro (Fig. 2f).

### Constitutive activation of NF-kB induced cell death and apoptosis

**Fig. 3:**
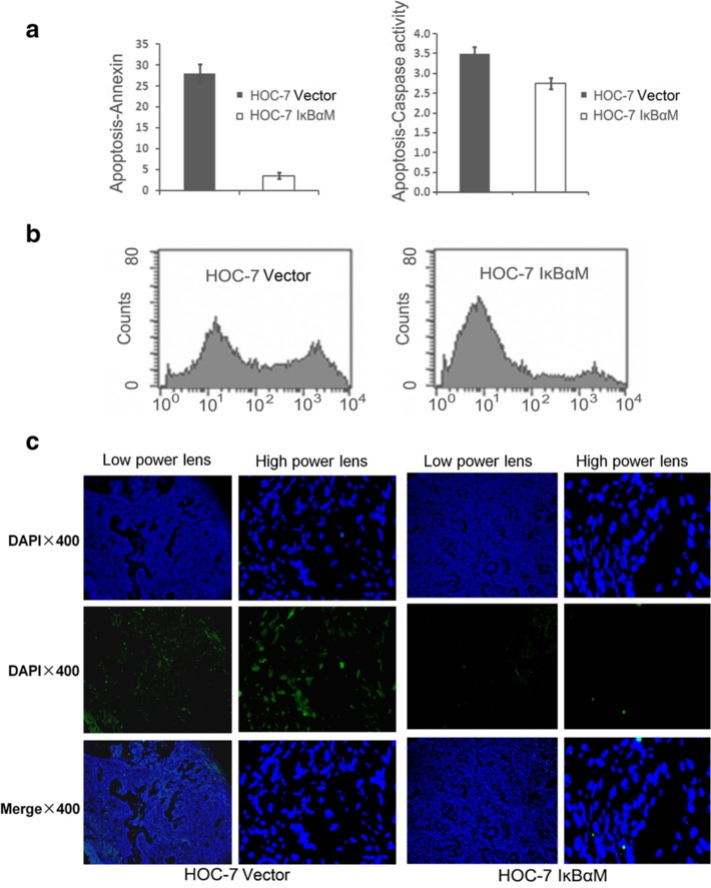
Tumor apoptosis following introduction of IkBαM in ovarian cancer cell lines. **a** Apopotic cells measured by Annexin V staining of cells with or without IkBαM expression. Error bars = 95 % confidence intervals. **b** Percentatge of apoptotic cells measured by flow cytometry. **c** representative images of xenograft tumor tissue showing apoptosis, which is indicated by DNA breaks (*green*) detected by an Apo-BrdU (*green*) in situ DNA fragmentation assay (×400, TUNEL). PI, red

**Fig. 4:**
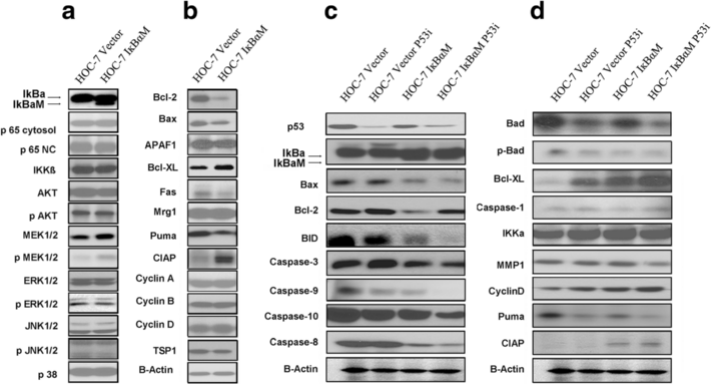
NF-kB - induced apoptosis in MAPKs independent pathway. **a** Cell apoptosis pathway are associated with inhibition of NF-kB activity. Detection of MAP kinases proteins in HOC-7 ovarian cancer cells with or without IkBαM by western blotting. **b** Detection of apoptosis-associated proteins in HOC-7 ovarian cancer cells with or without IkBαM. **c**-**d** NF-kB and p53 pathway related proteins in HOC-7 and relative transgenic cells by western blotting. β- actin was used as a loading control

To further evaluate the mechanisms involved in the transformation of ovarian epithelial cells mediated by IkBαM, we examined apoptosis in HOC-7 IkBαM and its parental cells, as detected by Annexin V staining, and in xenograft mouse tissures, as measured by an Apo-BrdU-labeled in situ DNA fragmentation assay (Fig. 3a-c). The incidence of apoptotic reduced 86 % in HOC-7 IkBαM cells compared with its respective parental cells. These data indicate that block of NF-kB reduced the levels of cellular apoptosis in HOC-7 cells. Western blot analysis cellular apoptosis pathways and indicated up-regulation of anti-apoptotic proteins including Bcl-xL, the reduction of pro-apoptotic proteins Bax, Bad, and Bid, and sequentially, reduction the activation of caspases 3, 8, 9 and 10 (Fig. 4b-d). The level of the anti-apoptotic protein Bcl-xL was increased in SKOV3/IkBαM cells compare with parental cells. Interesting, no changes in the level of Bcl-2 were visualized in SKOV3/IkBαM cells, whereas the Bcl-2 dramatically decreased in HOC-7 cells transfected with IkBαM (Fig. 4c-d). We reasonable doubt BCL-xl is the key protein involved in the phenotypic shift. Data showed that HOC-7 IkBαM cells over expressed BCL-xl compared with parental cells. The downstream factors such as caspase-3 and caspase-9 showed decrease expression. On the contrary, HOC-7 IkBαM cells carrying BCL-xl siRNA indicated increased expression of downstream factors, such as casapse-3 and casapse-9 (Fig. 2c). The results demonstrated that the NF-kB-mediated tumor suppression may be related to Bcl-xL, and this crucial marker change is keep consistent in the five independent ovarian cancer cell lines (SKOV3, HOC-7, HEY, OVCA433 and OVCR3) although they may have different genetic backgrounds [31].

### NF-kB - induced apoptosis correlated with MAPKs pathway

To study the role of Ras and MAPKs in human ovarian tumorigenesis, we compared the expression of signaling molecules involved in the mitogen-activted protein kinase (MAPK) pathway between KRAS mutant cell line HOC-7 and the KRAS wild type cell line SKOV3. Cells expressing IkBαM showed marked hyperplasia in either HOC-7 or SKOV3 transgenic cells, indicating that the blockage of NF-kB induce tumor proliferation independent on the mutational status of RAS/RAF. In nude mice, both of transgenic cell lines showed tumor inducing function. All together, In contrast with the result presented by others that Ras and NF-kB in a setting more relevant to human tumorigenesis, NF-kB inhibit the tumor growth in a Ras-independent way. Interestingly, western blotting showed high expression of p-MEK in the HOC-7 IkBαM cells, no changes were seen in MAPKs downstream pathway, including the JNK, ERK and p38 sub pathways (Fig. 4a). On the contrary, according to our previous data published, in SKOV3-IkBαM cell, dephosphorylation of ERK was observed as the upstream of mitochondria apoptosis pathway, suggesting that NF-kB induced apoptosis is associated with phosphorylation of mitogen-activated protein kinases in KRAS wild type cell line SKOV3 but not in KRAS mutation cell line HOC-7 which expression persistent activity of KRAS-MAPKs pathway [31].

### Nuclear localization of NF-kB is associated with favorable prognoses for ovarian cancer patients

**Fig. 5:**
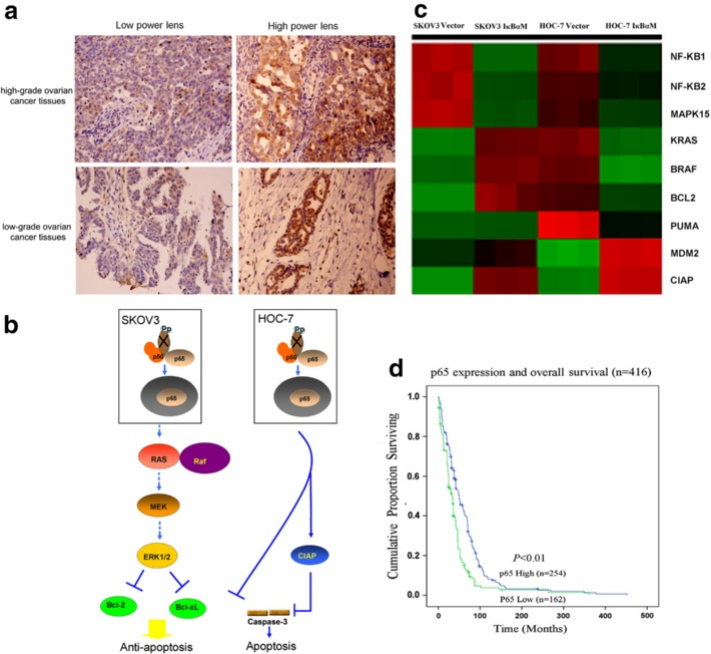
Correlation of nuclear localization of p65 NF-kB and clinicopathologic characteristics and a model of the NF-kB role in ovarian cancer. **a** Representative images showing high and low nuclear expression of p65 in high-grade ovarian cancer tissues. **b** A model illustrating that NF-kB suppresses tumorigenesis by inhibiting anti-apoptosis through the regulation of MAPK phosphorylation and apoptosis pathway. **c** Detection of NF-kB1, NF-kB2, MAPK15, KRAS, BRAF, PUMA, MDM2, CIAP by heatmap. **d** Kaplan-Meier curves showing favorable overall survival rates in patients with low-grade serous ovarian cancer are associated with nuclear expression of p65

Although NF-kB was reported to be a poor prognostic indicator in ovarian cancer, our study suggested that NF-kB may function as a tumor suppressor in low-grade ovarian cancer. Therefore, we further analyzed whether nuclear NF-kB p65 expression predicts prognosis in patients with low-grade ovarian serous carcinoma by immunostaining a tissue microarray consisting of 416 low-grade ovarian cancer cases. We found that the nuclear expression of p65 was low (<12 %, nuclear positive tissues) in 38.9 % of cases (162/416) but high (≥12 %, nuclear positive tissues) in 61.1 % of cases (254/416). We found a significant statistical correlation between cases with low and high expression of nuclear p65 and their prognostic (*P* < 0.01). Patients with high nuclear expression of p65 had longer overall survival than did patients with low p65 expression (Fig. 5d). The 5-year survival time was 21 months longer in cases with high NF-kB p65 nuclear expression than in cases with low NF-kB p65 nuclear expression. Furthermore, the cumulative proportions of patients surviving 24, 60, and 120 months were greater among patients with high nuclear p65 expression than patients with low nuclear p65 expression (0.755, 0.427,0.081 vs 0.623, 0.164, 0.038). Images representing patient tumor tissues with high and low nuclear expression of p65 are shown in Fig. 5a. These results suggest that NF-kB nuclear activation is associated with outcome of disease in patients with ovarian cancer.

### Crucial pathways that involved in low-grade papillary serous ovarian carcinoma growth due to inhibition of nuclear factor-kappa B

The heatmap displays all genes that were differentially expressed between these cells (Fig. 5c), in which red or green colors indicate up-or down regulated genes, respectively. The NF-kB1, NF-kB2 and MAPK15 genes expression were obviously decreased in ovarian cancer cell lines with dominant negative IkBαM. Furthermore, comparing SKOV3 with SKOV3 IkBαM, apoptosis relative genes such as PUMA were decreased and proliferation relative gene CIAP and MDM2 were increased. Similar, comparing HOC-7 with HOC-7 IkBαM draw the same conclusion. Different with SKOV3 cell line, HOC-7 shows different change in MAPKs pathway factors including MAPK15, KRAS and BRAF. These results verified that NF-kB - induced apoptosis in MAPKs independent pathway. Furthermore, the expression of Bcl-2 is different between SKOV3 IkBαM and HOC-7 IkBαM cells [31]. As shown in Fig. 4d, even though mitochondrial pathway is the shared downstream of both SKOV3 and HOC-7, the data indicated that NF-kB reduced Bcl-2 expression and lead to apoptosis in SKOV3 cells depending on dramatically increased the levels of phosphorylated MEK1/2 and ERK1/2, whereas in HOC-7 cells, NF-kB directly affects downstream apoptosis pathway through CIAP and caspase-3 and get the same results eventually (Fig. 5b).

### NF-kB - induced apoptosis in p53 independent pathway

**Fig. 6:**
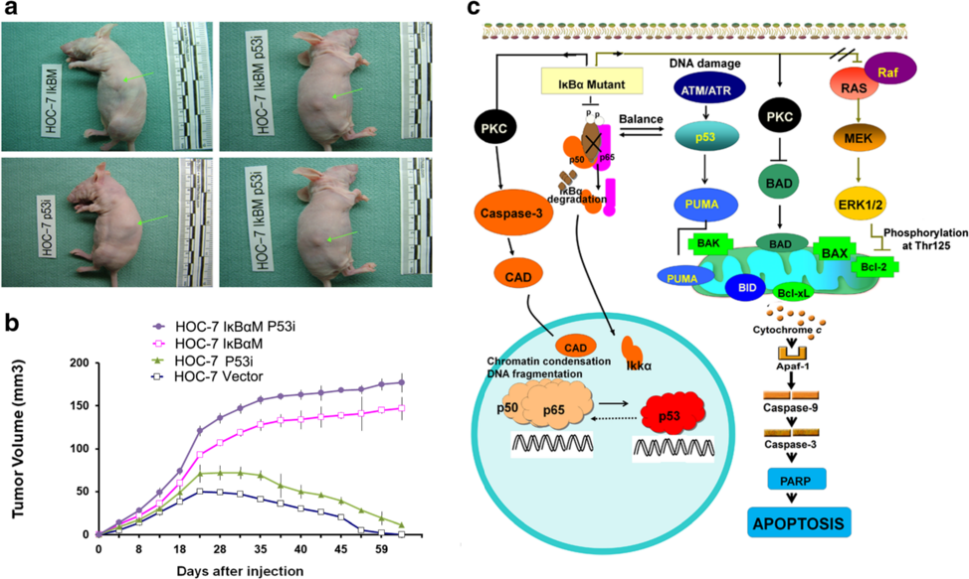
NF-kB induced apoptosis in p53-independent pathway in BALB/athymic nude mice. **a** Inhibition of NF-kB activity can induce tumor growth in p53-independent pathway in vivo. **b** Tumor growth following etopic expression of IkBαM in BALB/athymic nude mice in p53-independent pathway. **c** A model illustrating that inhibition of NF-kB signaling in ovarian tissue results in an increased proliferation rate and the development of ovarian tumors. Furthermore, neither mutations in the KRAS nor decreasing expression of p53 gene can affect the tumor development induced by IkBαM

In an attempt to elucidate the crosstalk between NF-kB and p53, we generated retrovirus expressing small interfering RNA (siRNA) against p53 in HOC-7 and HOC-7- IkBαM cell lines. Western blotting showed p53 expression was decreased after infection with p53i RNA. As described previously, we examined the cell proliferation, anchorage independent growth on soft agar of these cell lines carrying knock out p53, knock out NF-kB, double knock out NF-kB and p53, or parental cell. As shown in Fig. 2a-d, both HOC-7-IkBαM and HOC-7-IkBαM-p53i cells displayed dramatically increase of cell proliferation and colony formation as compared with parental cells. Surprisingly, we found that treatment of HOC-7 IkBαM cells with small interfering RNA against p53 reversed the decrease of pro-apoptotic protein Bcl-2, wheras no big changes in the level of anther apoptotic-related proteins Bcl-xL, caspase 8. caspase 9 and caspase 10. These results demonstrated that blocking NF-kB function in ovarian cancer cells may promote the proliferation by increasing the levels of cellular anti-apoptisis proteins Bcl-xL in p53 independent way. In order to verify above point of view in vivo, we first established xenograft models with HOC-7 IkBαM and HOC-7 IkBαM p53i cells using parental cells as control. As shown in Fig. 6a-b, nude mice injection with HOC-7 IĸBαM p53i had more subcutaneous tumor growth than those injected with HOC-7 IĸBαM cells. Similarly, nude mice carry HOC-7 p53i had more subcutaneous tumor growth than those injected with HOC-7 cells. All the changes are subtle. Decreasing expression of p53 gene will not affect the tumor development induced by IkBαM (Fig. 6c).

## Discussion

For a long time, the role of NF-kB was described as oncogeic with respect to its function in promoting cell proliferation, angiogenesis, transformation and invasion [9, 32–34]. A novel role for NF-kB has been suggested in several recent studies, specifically, involvement of NF-kB in tumor suppress has been demonstrated in skin and liver [35–37]. It seems NF-kB can either promote or inhibit carcinogenesis depending on the tumor type or cell specialty. Because therapeutics inhibiting NF-kB pathway are being used as a strategy in cancer treatment, it is crucial to explore potential mechanism and altering function of NF-kB that can protect or contribute to apoptosis. In this study, we characterized an ovarian cell line derived from a well-differentiated (low grade) serous carcinoma and defined a tumor suppressor role of NF-kB in tumor development. We demonstrated that introduction of a dominant negative mutant IĸBα (IĸBαM), which can firmly binding with NF-kB and inhibit NF-kB translocation, resulted in cell proliferation and inhibition of apoptosis in low grade ovarian epithelial cancer in vitro and in vivo.

Ras, the important genes in tumorigenesis, were found to contain activating point mutations in human low grade ovarian epithelial carcinoma cell line HOC-7 [8]. The mutations of KRAS may initiate the development of additional genetic “hit” in tumorigenesis [38, 39]. Since the mutations of KRAS are early events involved in tumor initiation, HOC-7 provide a good model to explore the connection between ras-raf-MAPKs pathway and NF-kB in tumorgenesis [6]. HOC-7 cell lines have the KRAS mutation, presumably keeping activated the MAPKs pathway, showed phosphorylation of MEK after infection of IkBαM, while SKOV3 cell line, which sequenced to be the KRAS wild type, showed dephosphorylation of both MEK and ERK following the infection of IkBαM. No matter MAPK pathway activated or not, both transgenic cell line SKOV3- IkBαM and HOC-7- IkBαM showed potential tumorigenesis potent both in vitro and in vivo. All together, the IkBαM construct in cancer cell can induce the tumor growth in a ras independent way. NF-kB can serve as a tumor suppressor through its pro-apoptotic function in ovarian cancer cells in Ras-MAPKs independent pathway.

Accumulating evidence shows that nuclear function of IkBα is its participation with NF-kB in an autoregulatory negative feedback loop culminating in the translocation of NF-kB into the nucleus. The recent identification showed that phosphorylation of CBP by Ikkα can switch the binding reference of CBP from p53 to NF-kB, which provided an important link between p53 and NF-kB [40–42]. Furthermore, Ikkα up-regulates the expression of p53 antagonist MDM2, which can dominantly localized in the nucleus and promotes increased degradation of p53 [41–43]. Since Ikkα was regarded to release upon phosphorylation IkBα, it was therefore postulated that IkBα maybe affect the balance between p53 and NF-kB. In our study, the HOC-7 p53i showed overexpression of Ikkα, implied the increase activity of NF-kB [44, 45]. In parallel with these results, we also found that HOC-7 IkBα decreased the expression of NF-kB responsive promoter CIAP and increased those with p53-responsive promoter (p21 and mdm2). All of above suggested that Ikkα and downstream IkBα regulated the antagonism crosstalk between NF-kB and p53. To further examine if p53 paly a possible role in the development of transgenic tumors, we set up the xenograft model of HOC-7-53i, HOC-7-IkBαM and HOC-7-IkBαM-p53i. We observed an increased tumor growth in HOC-7 p53i, suggestion an involvement of p53 in the development of these tumors, which consist with previous knowledg.e that the tumor suppressor p53 inhibits cell growth through activation of cell-cycle arrest and apoptosis. Mutation analysis of exons 5–8 of p53 in HOC-7 parental and transfected cells did not reveal any mutation. Kevin M showed that induction of p53 causes an activation of NF-kB which correlates with the function of p53 to induce apoptosis [46]. Inhibition of NF-kB activity abrogated p53-induced apoptosis, indicating that NF-kB is essential in p53-mediated cell apoptosis. Loss of NF-kB activity specifically abrogated the p53-mediated apoptosis response. Activation of NF-kB by p53 was different from that mediated by tumour-necrosis factor-a and involved MEK1 and the activation of pp90rsk. Inhibition of MEK1 blocked activation of NF-kB by p53 and completely abrogated p53-induced cell death. Our studies showed knocking down p53 augment tumor growth in HOC-7 IkBαM cells, suggesting that NF-kB is crucial but not cover all part in p53-mediated cell apoptosis.

## Conclusions

The results indicated a new role of NF-kB in the inhibition of tumor growth. Inhibition of NF-kB signaling in ovarian tissue results in an increased proliferation rate and the development of ovarian tumors. Furthermore, neither mutations in the KRAS nor decreasing expression of p53 gene can affect the tumor development induced by IkBαM [47]. The drugs used targeted the NF-kB, where p53 is itself an important mediator of chemosensitivity, such therapy seems very dangerous to the tumor treatment due to its repression of p53-mediated tumor cell death function. Liu demenstrated that NF-kB can be reprogrammed to a tumor-promoting oncogene conferring drug resistance following standard chemotherapy with carboplatin and paclitaxel, clinical treatment of ovarian cancer using anti-NF-kB agents should be cautiously considered and guided by specific markers that can distinguish whether NF-kB is functioning as a tumor suppressor or an oncogene. Comparing Bcl-xL expression between primary and recurrent ovarian tumors after chemotherapy might be a useful marker to determine the function of NF-kB and direct clinic individualized drug therapy.

## Abbreviations

CMV, cytomegalovirus; EMSA, electrophoretic mobility shift assay; HCC, hepatocellular carcinoma; MAPK, mitogen-activated protein kinase; SCCs, squamous cell carcinoma
